# Maternal tobacco use during pregnancy and risk of congenital heart defects in offspring: a systematic review

**DOI:** 10.1186/1617-9625-12-S1-A20

**Published:** 2014-06-06

**Authors:** Aspasia Tzani, Konstantinos P Economopoulos

**Affiliations:** 1 Society of Junior Doctors, Athens, 15123, Greece; 2 Medical school, National and Kapodistrian University of Athens, Athens, 115 27, Greece; 3 Massachusetts General Hospital, Harvard Medical School, Boston, Massachusetts, 02114, USA

## Background

Numerous human studies have investigated potential teratogenic effects of maternal smoking during pregnancy, with conflicting findings [1,2]. One of the most common birth malformations is congenital heart defect (CHD) with a prevalence of around 1% of live births [3,4]. More specifically, several studies indicate that maternal tobacco use is related with increased risk of CHD, whereas other studies do not find any association [5,6]. The aim of this study is to systematically review the literature regarding the correlation between maternal smoking during pregnancy and the prevalence of CHDs in offspring.

## Material and methods

A thorough up-to-date search of the English literature through PubMed identified studies of maternal smoking during pregnancy and CHD in infants. The selected Mesh terms included ”maternal”, “tobacco”, “smoking”, ”cigarette”, “pregnancy”, “cardiovascular”, “abnormalities” ”congenital abnormalities”, “congenital heart defect”, ”offspring”. The main parameters that were extracted from the eligible studies were maternal characteristics (age, weight, race, socioeconomic and health status), smoking habits (cigarettes per day, fetal exposure period) and CHD subtypes.

## Results

The systematic search identified 874 articles, of which 37 met the inclusion criteria. Most studies have shown a strong correlation (OR: 2.06; 95% CI: 1.20–3.54) between mothers who reported medium and heavy smoking (≥1 pack per day) during the first semester of pregnancy and infants with septal heart defects than women who did not smoke during this time period. Maternal age, weight and socioeconomic status are independent confounding factors for neonatal heart defects. Women who smoked ≥25 cigarettes per day had increased risk to have infants with right-sided obstructive defects compared with nonsmoking mothers. Infants with CHD were more likely to be premature and have lower birth weight than healthy infants.

## Conclusions

Maternal cigarette smoking during pregnancy is associated with congenital heart defects, especially with septal and right-side obstructive defects [7]. Additionally, future studies should take place in order to understand all underlying mechanisms and set the starting point of population-based prevention strategies so as to encourage more women to quit smoking before or early in pregnancy resulting in decreased infant mortality and morbidity.

## References

[B1] Kallen K Maternal smoking and congenital heart defects Eur J Epidemiol 1999 15 731 737 10.1023/A:1007671631188 10555617

[B2] Karatza AA Giannakopoulos I Dassios TG Belavgenis G Mantagos SP Varvarigou AA Periconceptional tobacco smoking and isolated congenital heart defects in the neonatal period Int J Cardiol 2011 148 295 299 10.1016/j.ijcard.2009.11.008 19951824

[B3] Yerushalmy J Congenital heart disease and maternal smoking habits Nature 1973 242 262 263 10.1038/242262a0 4696256

[B4] Hackshaw A Rodeck C Boniface S Maternal smoking in pregnancy and birth defects: a systematic review based on 173,687 malformed cases and 11.7 million controls Hum Reprod Update 2011 17 589 604 10.1093/humupd/dmr022 21747128 PMC3156888

[B5] Ferencz C, Rubin JD, Loffredo CA, Magee CA Perspectives in pediatric cardiology. Epidemiology of congenital heart disease. The Baltimore-Washington Infant Study 1981–1989 4 New York: Futura Pub, Mount Kisco 1993 last page

[B6] Kuciene R Dulskiene V Maternal socioeconomic and lifestyle factors during pregnancy and the risk of congenital heart defects Medicina Kaunas 2009 45 904 909 20051723

[B7] Malik S Cleves MA Honein MA Romitti PA Botto LD Yang S Hobbs CA National Birth Defects Prevention Study: Maternal smoking and congenital heart defects Pediatrics 2008 121 e810 e816 10.1542/peds.2007-1519 18381510

